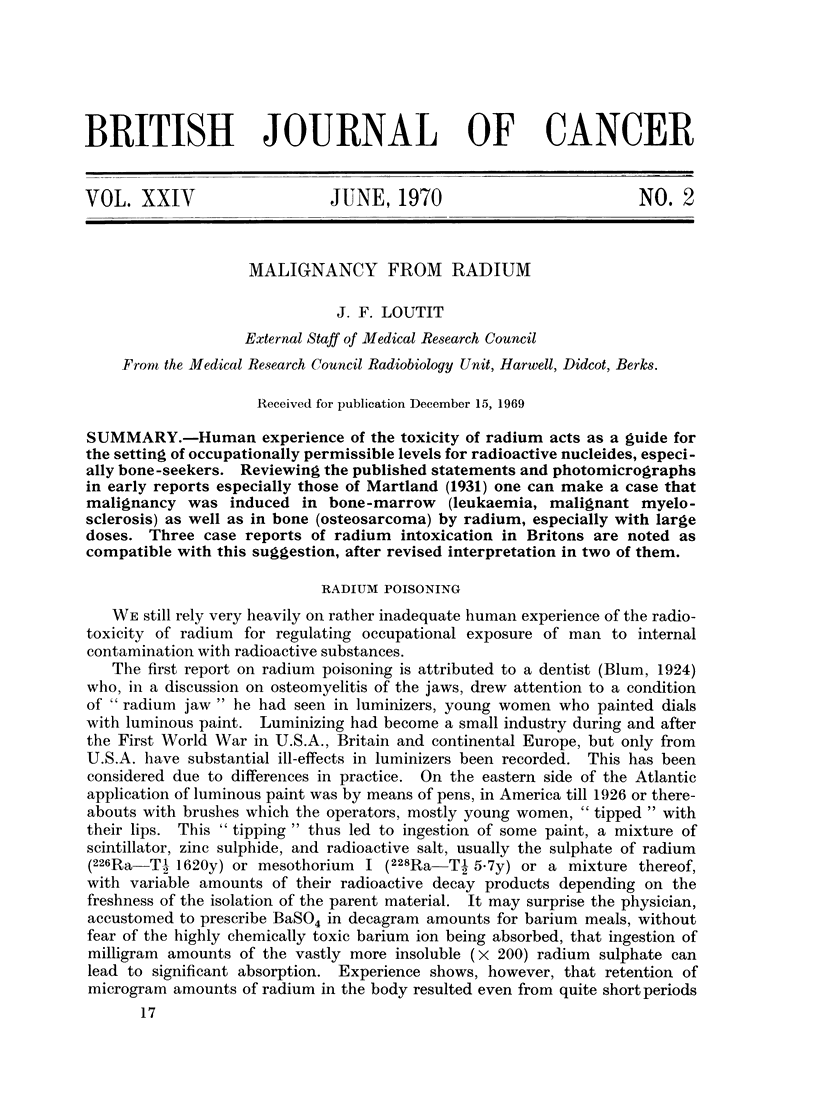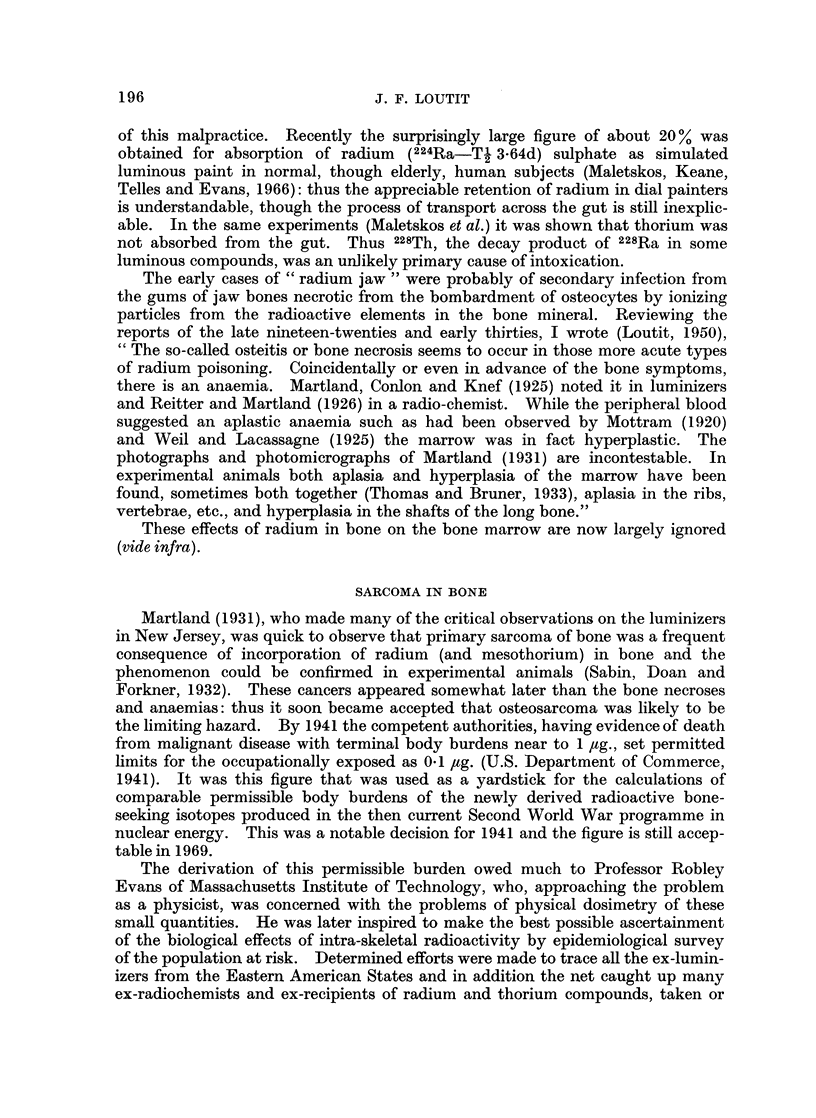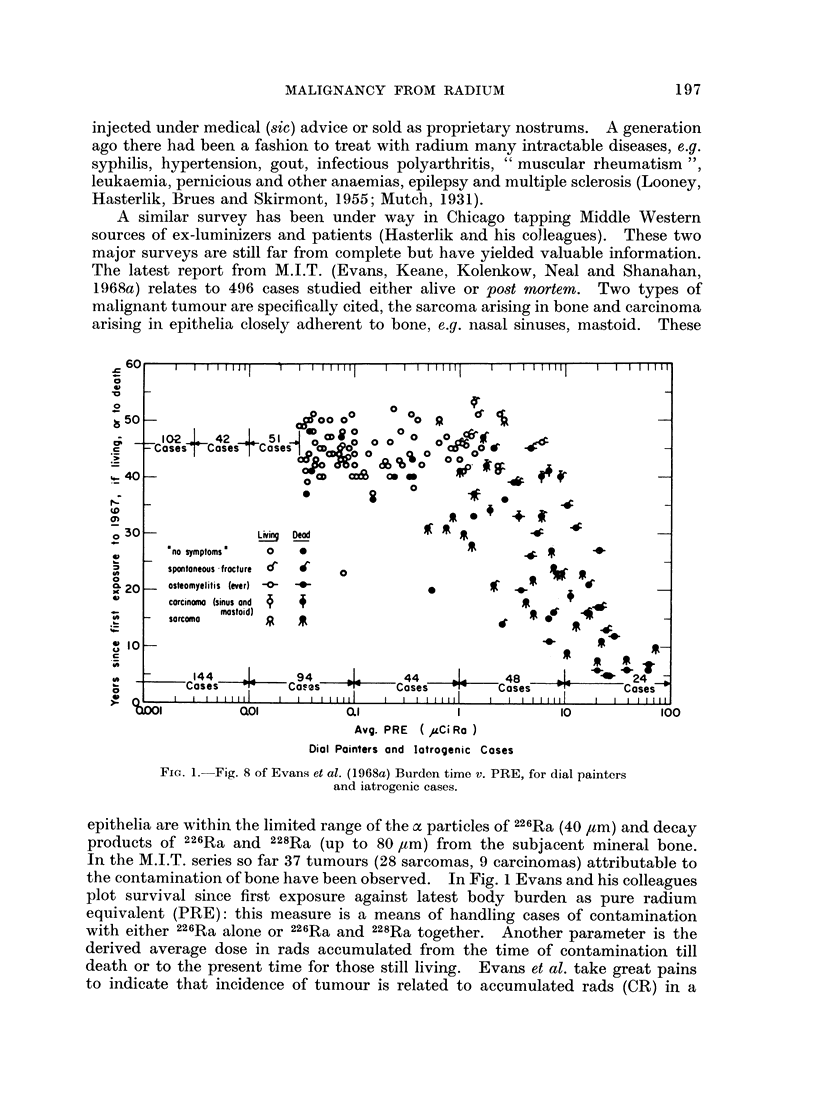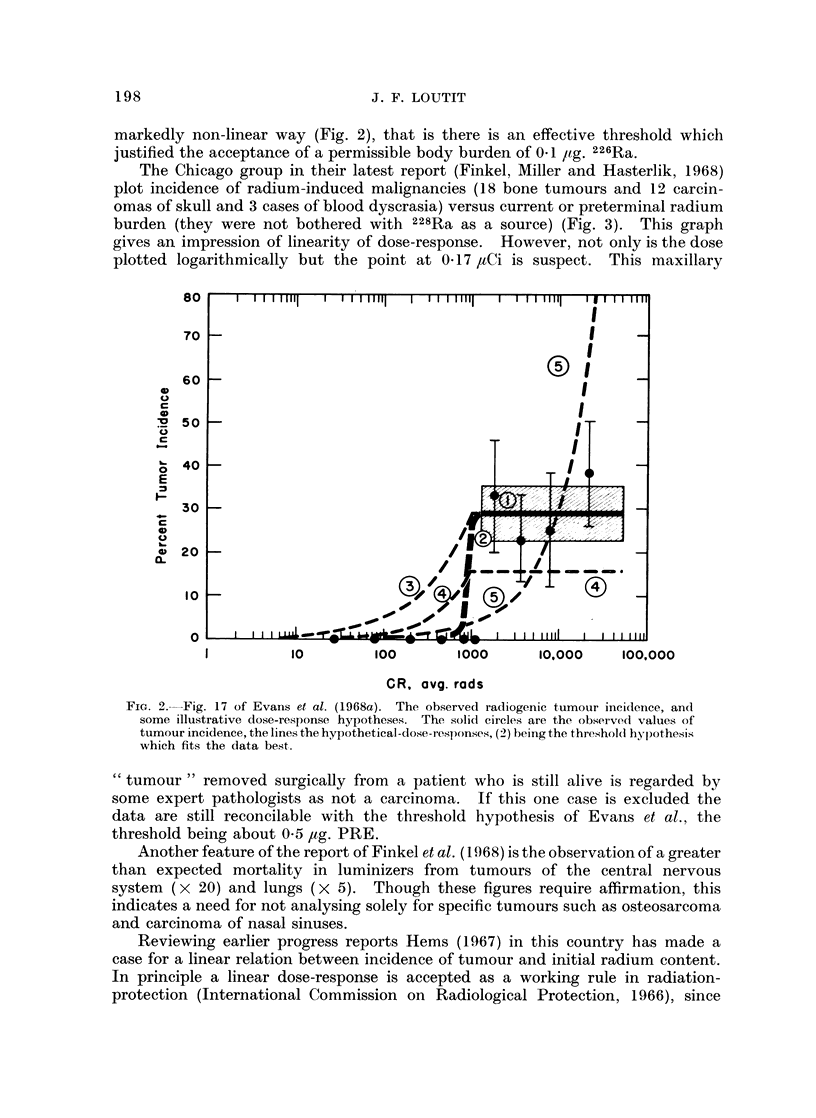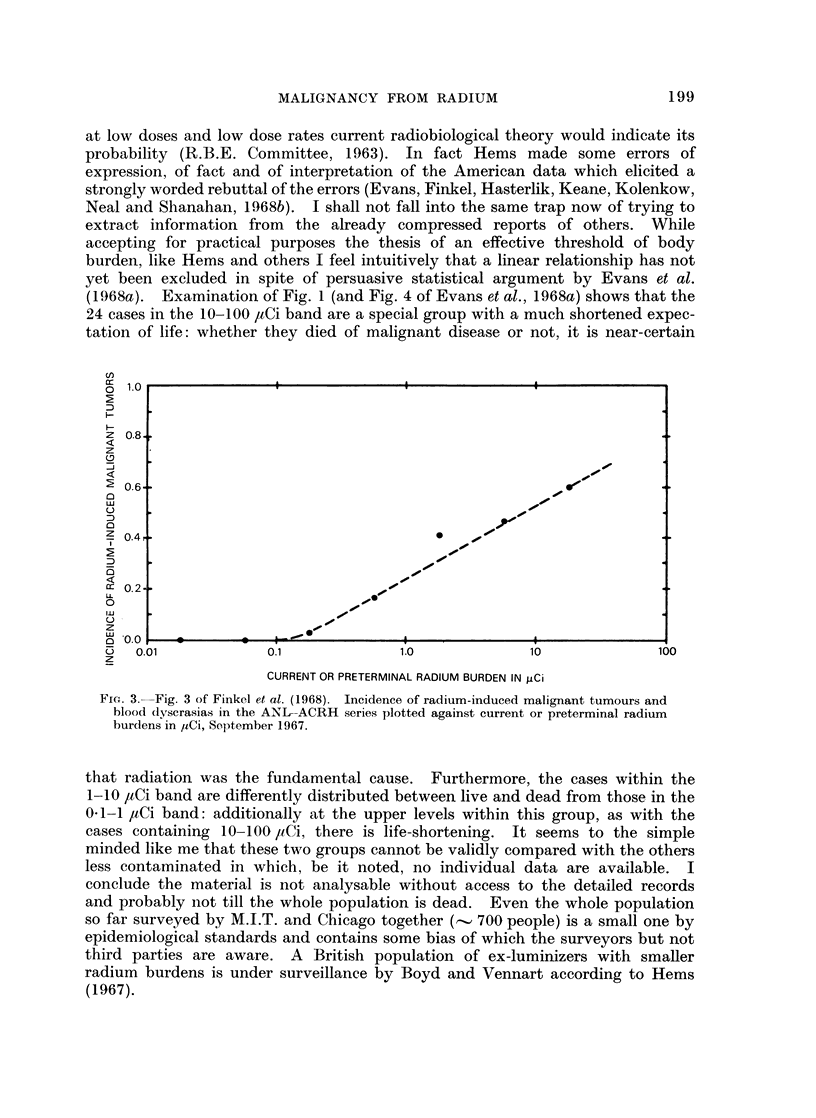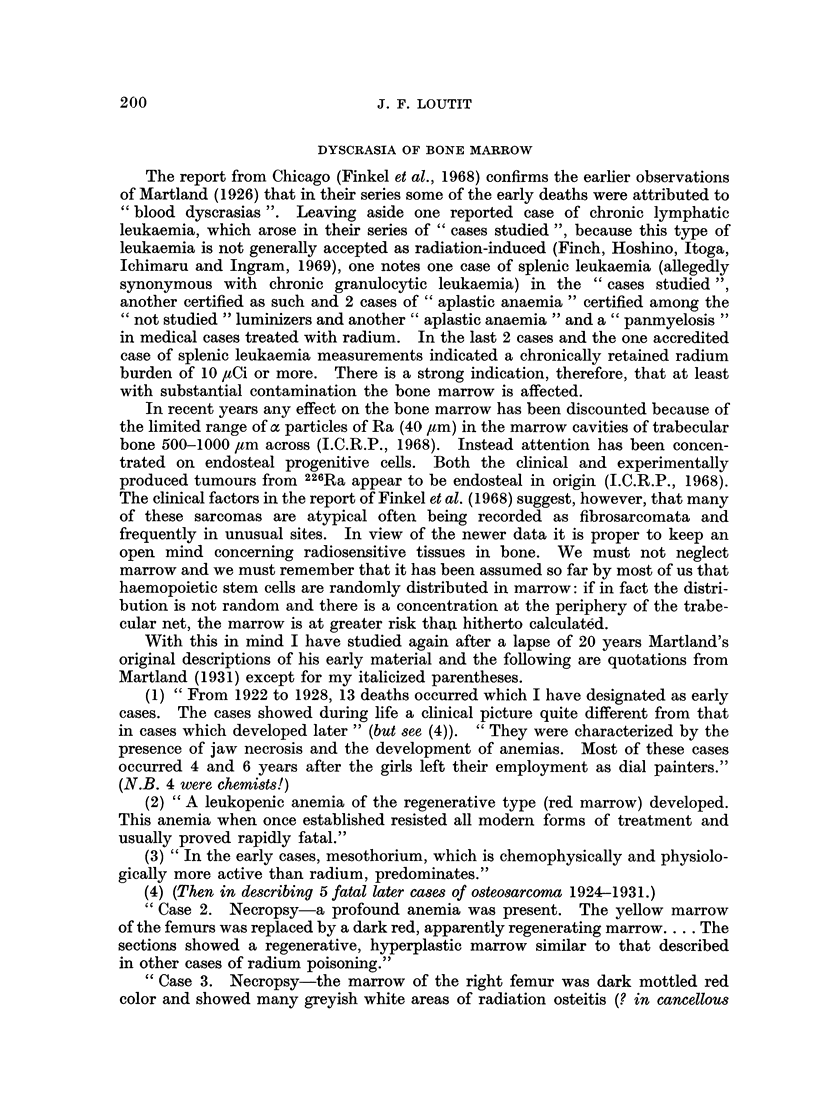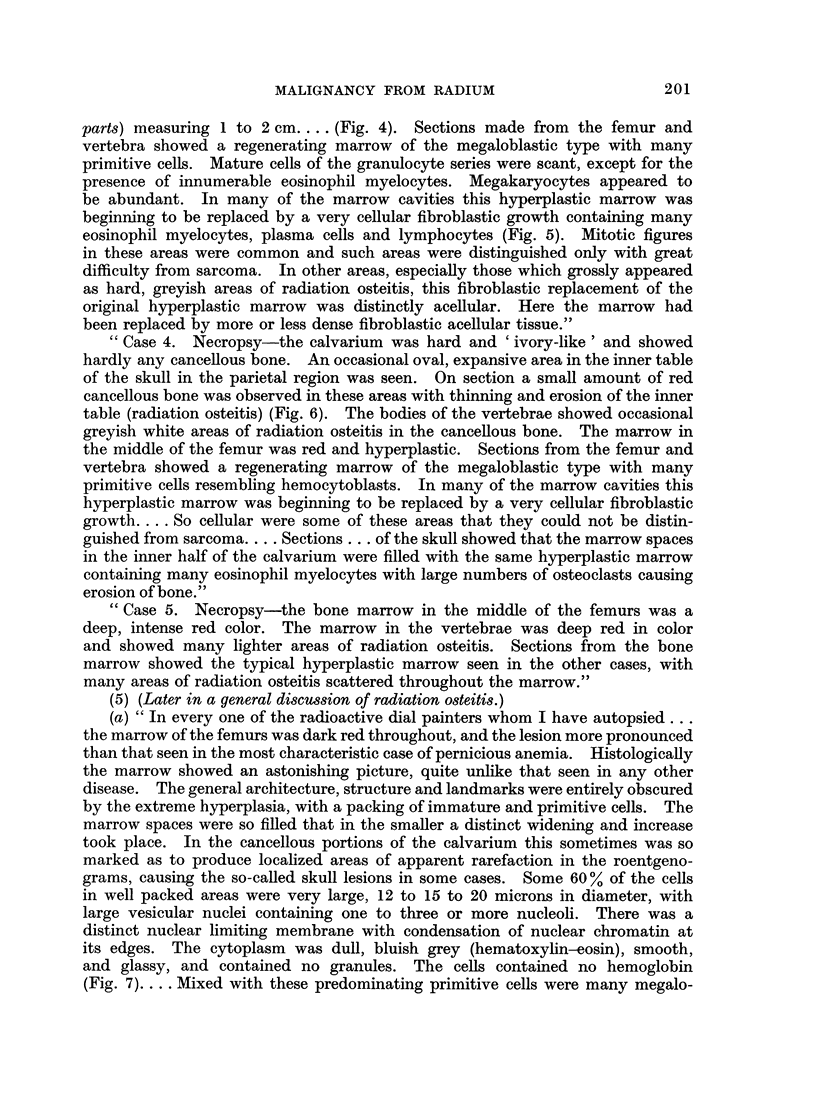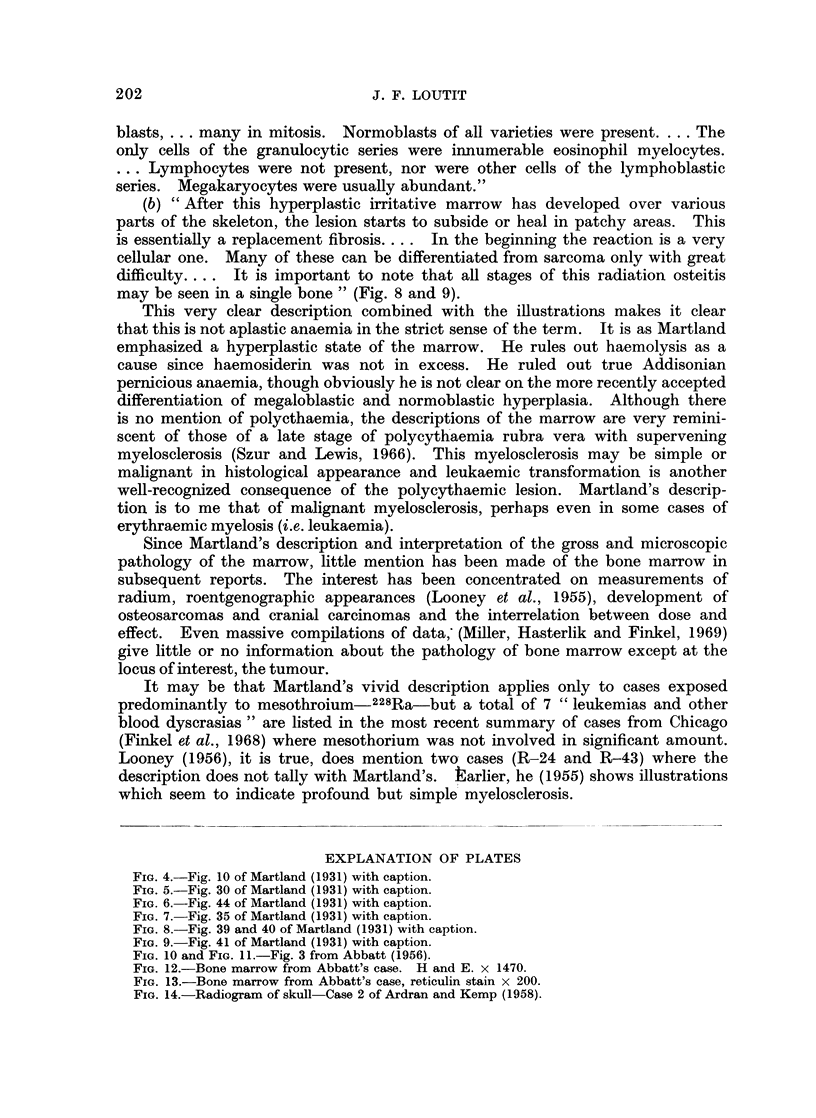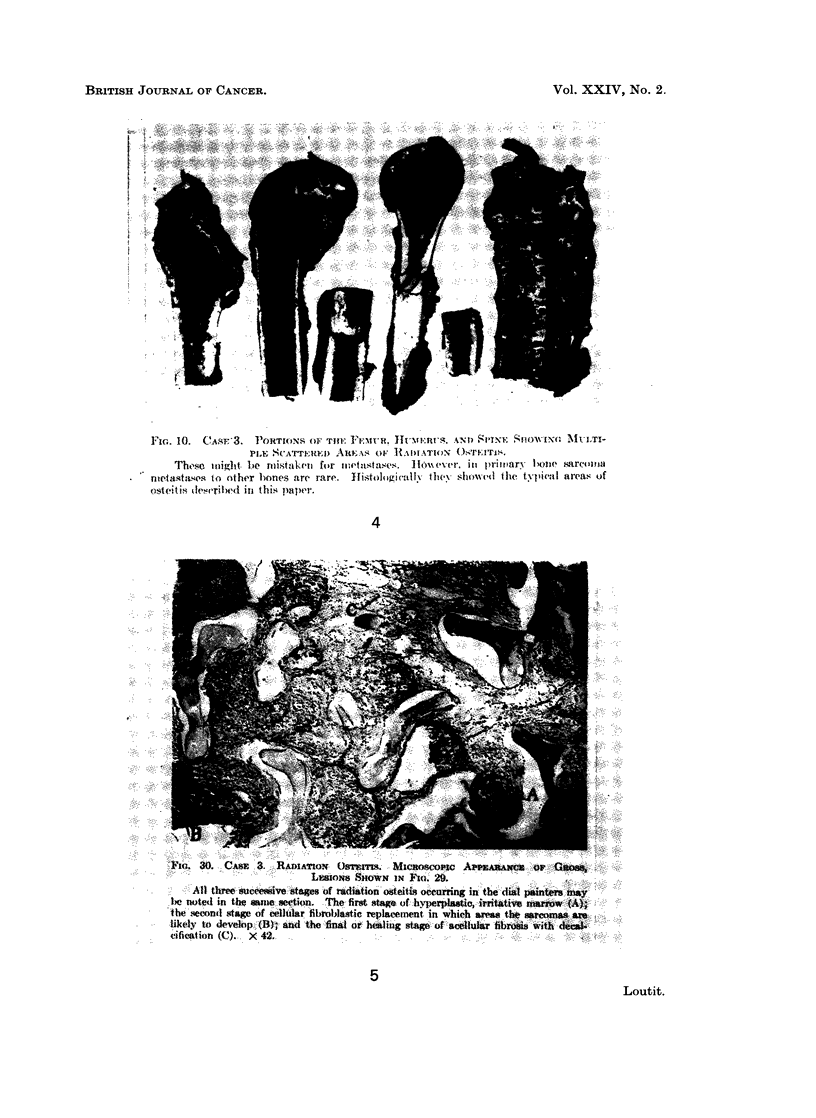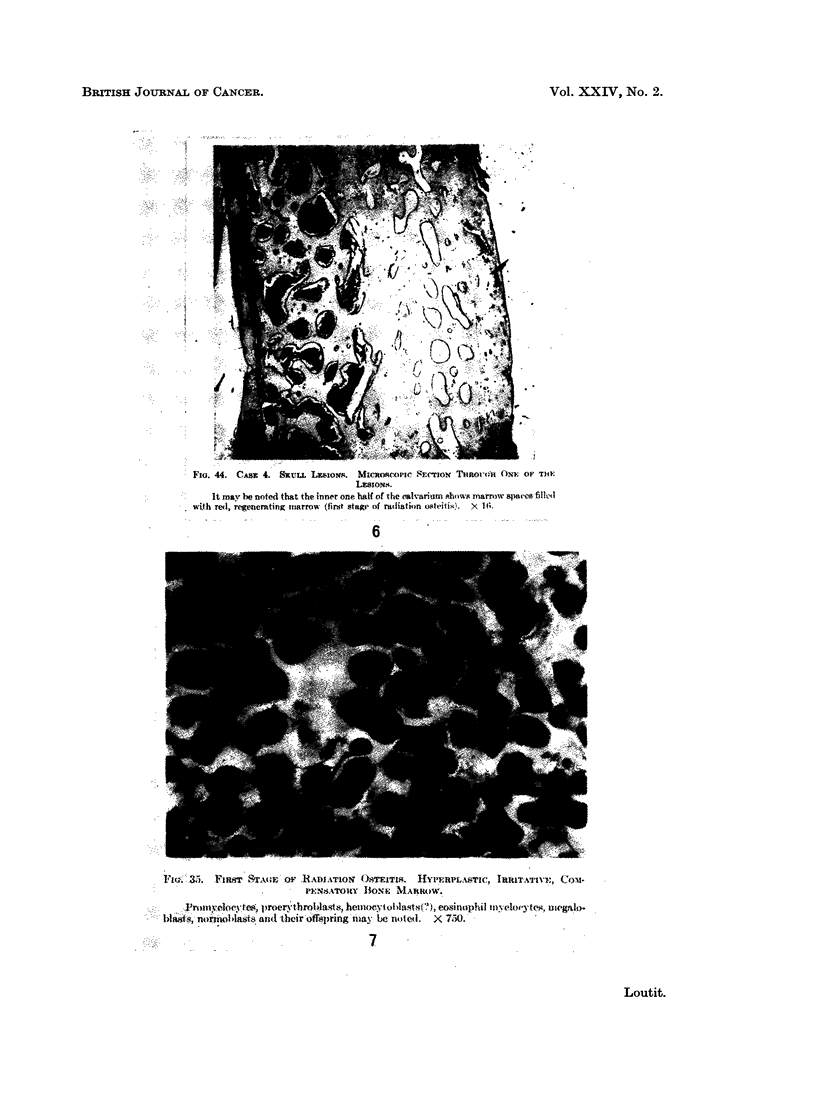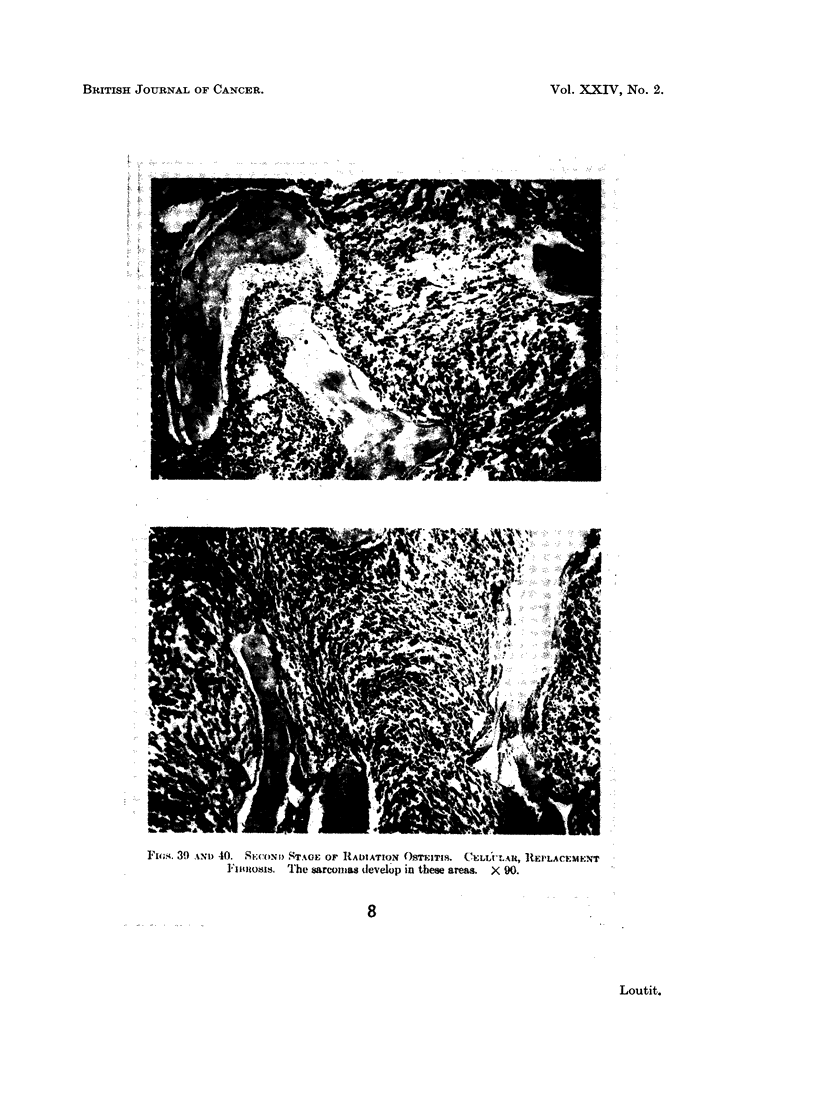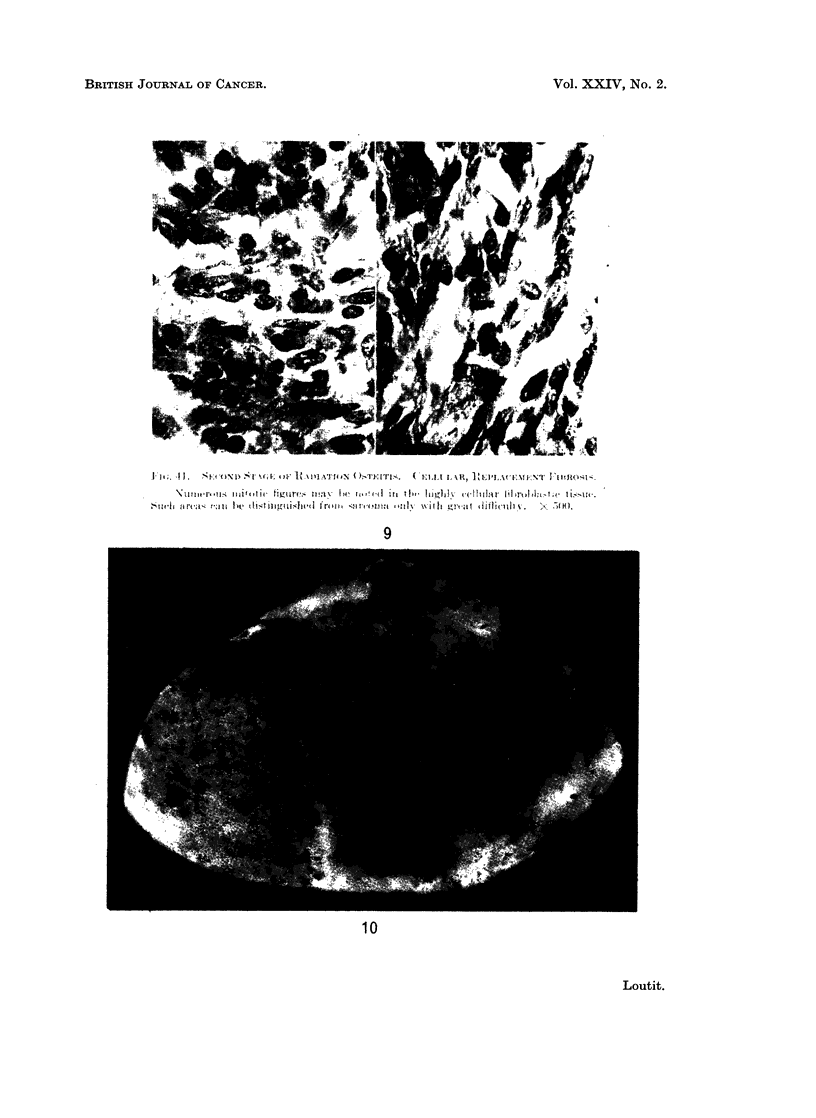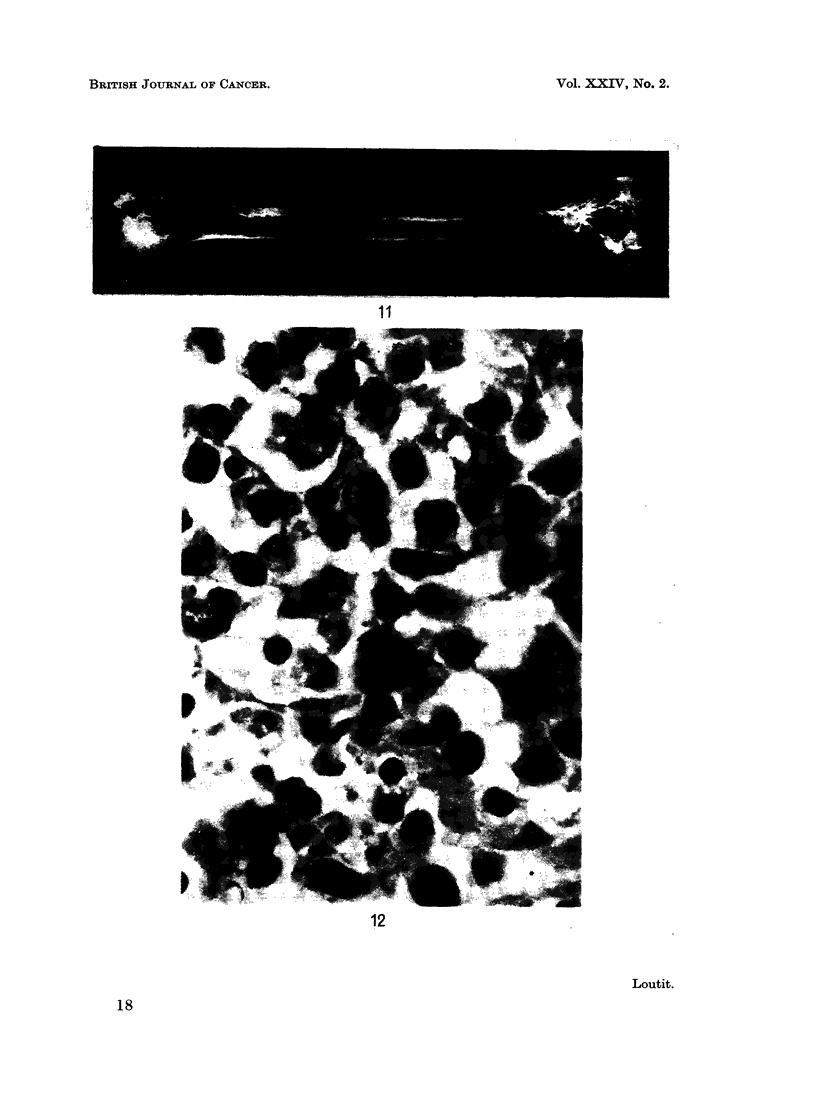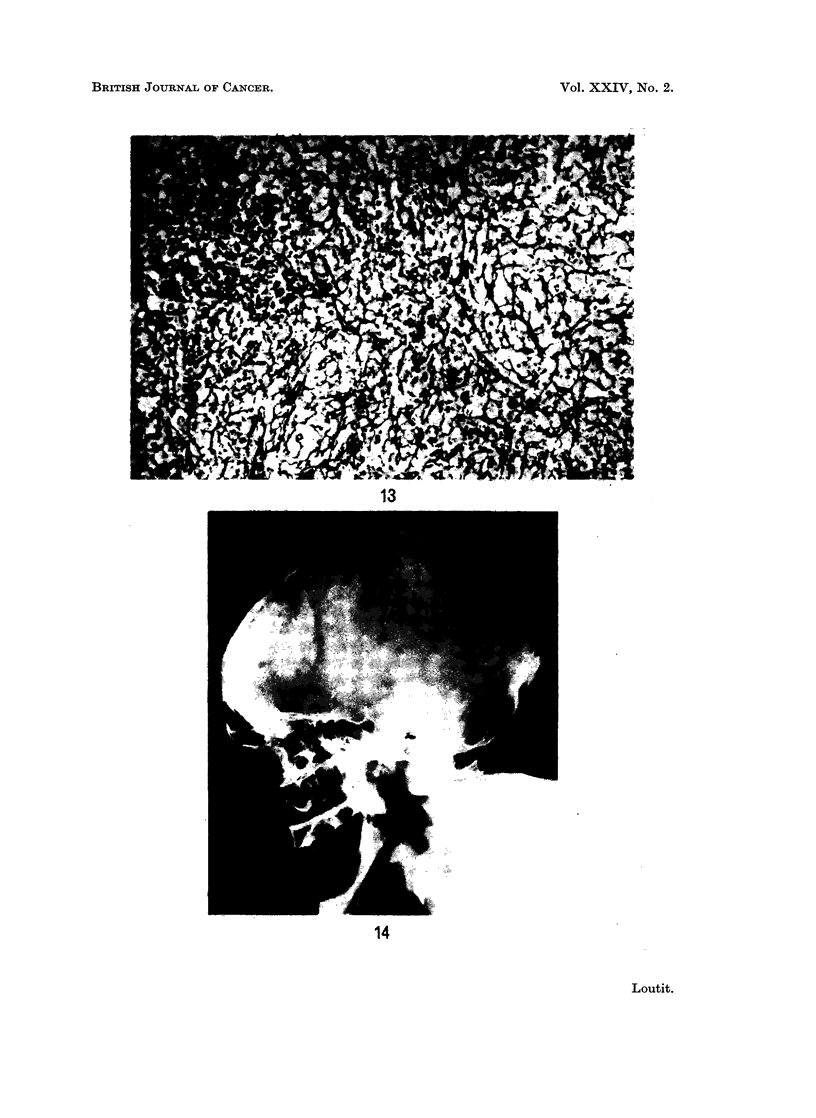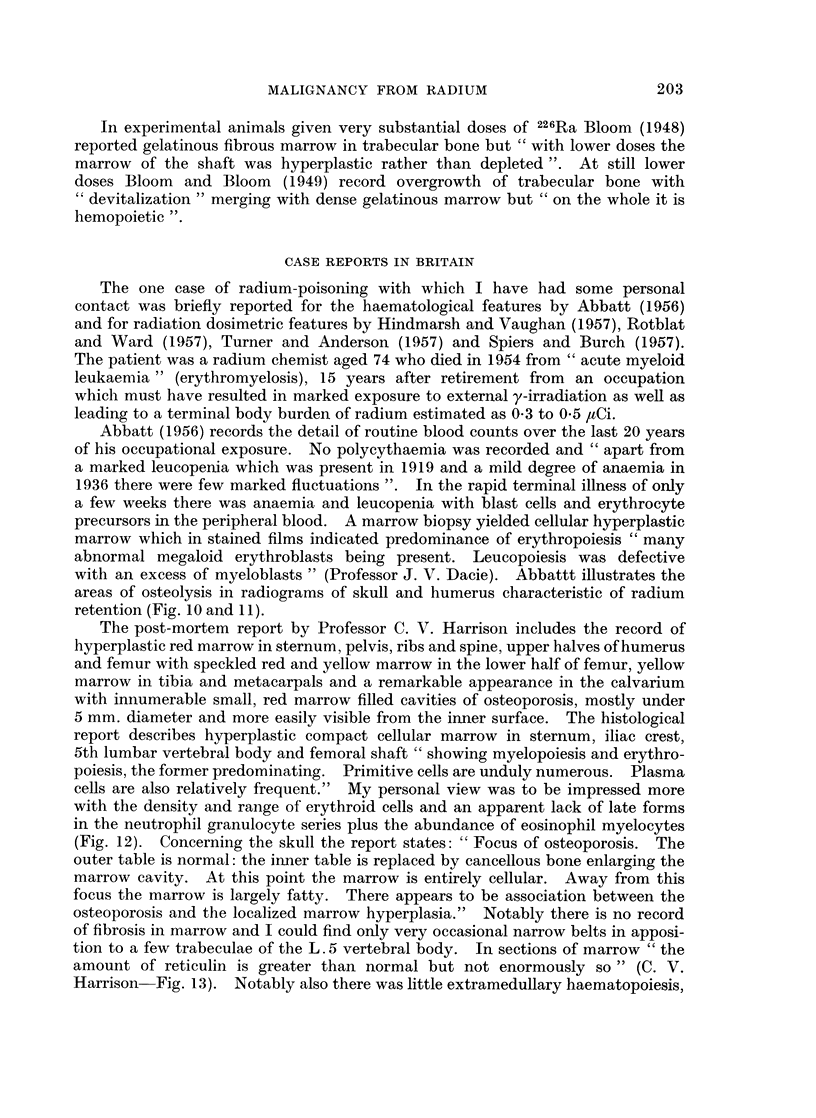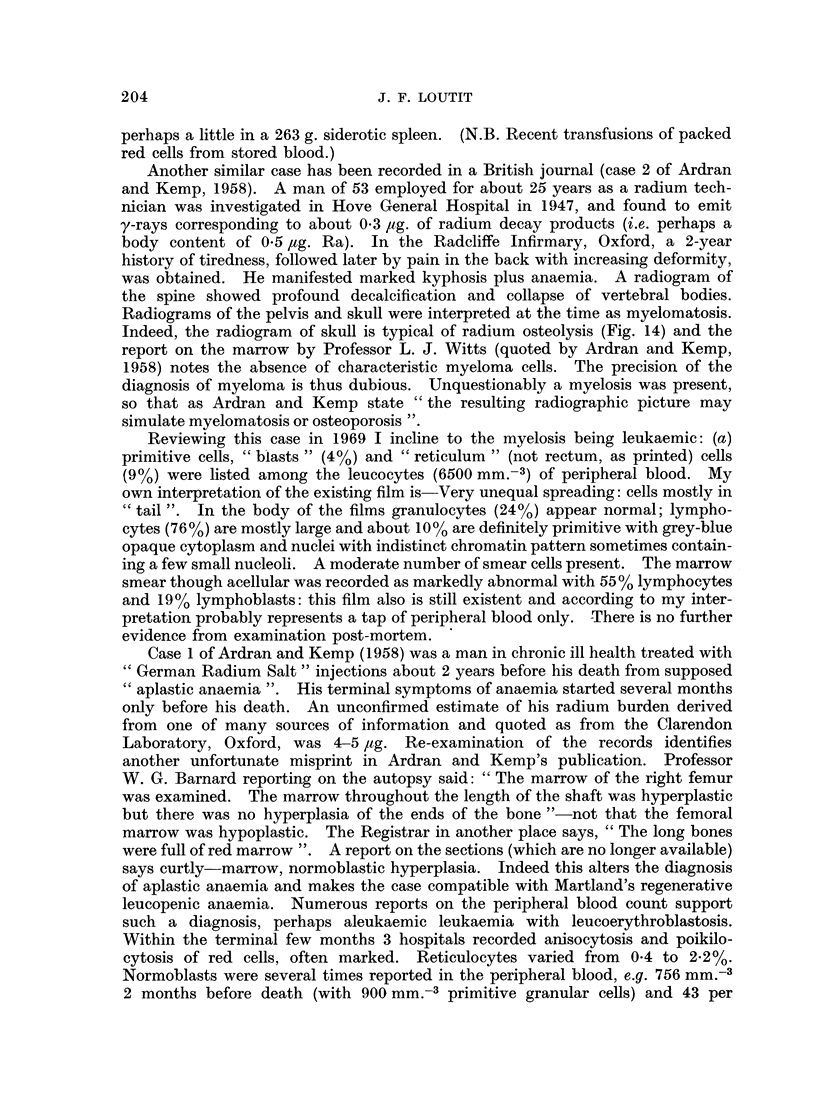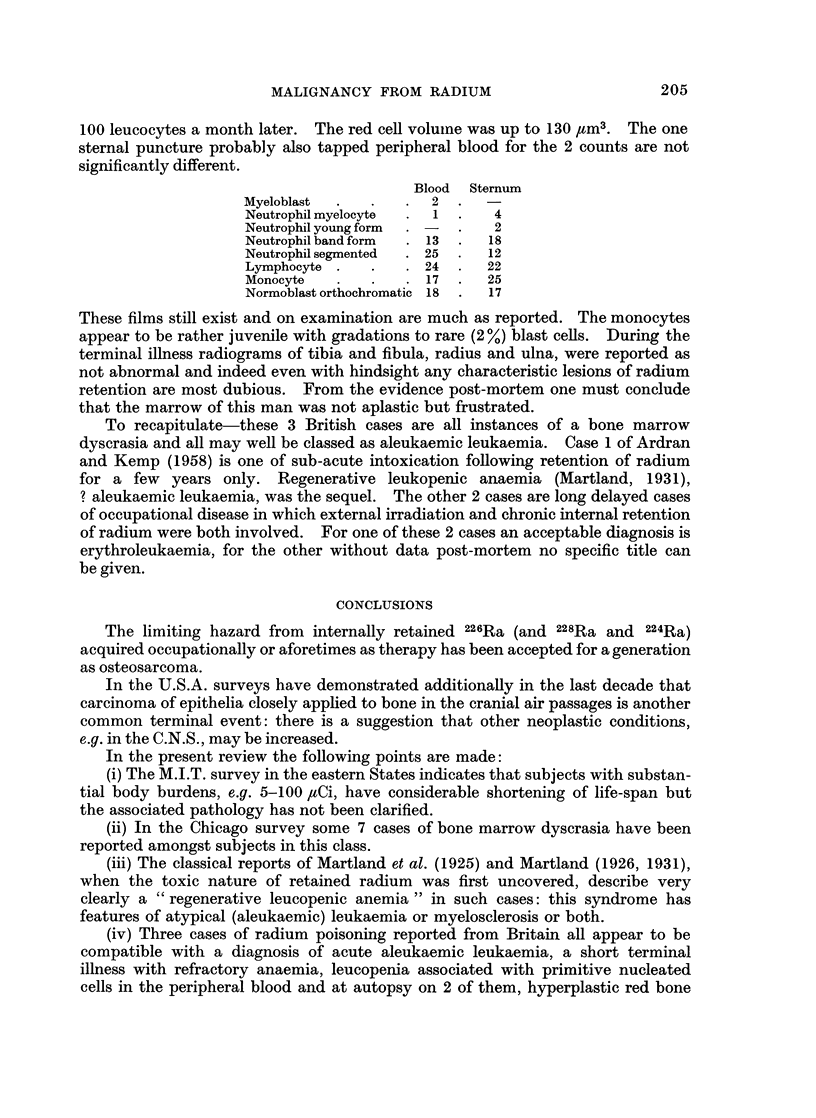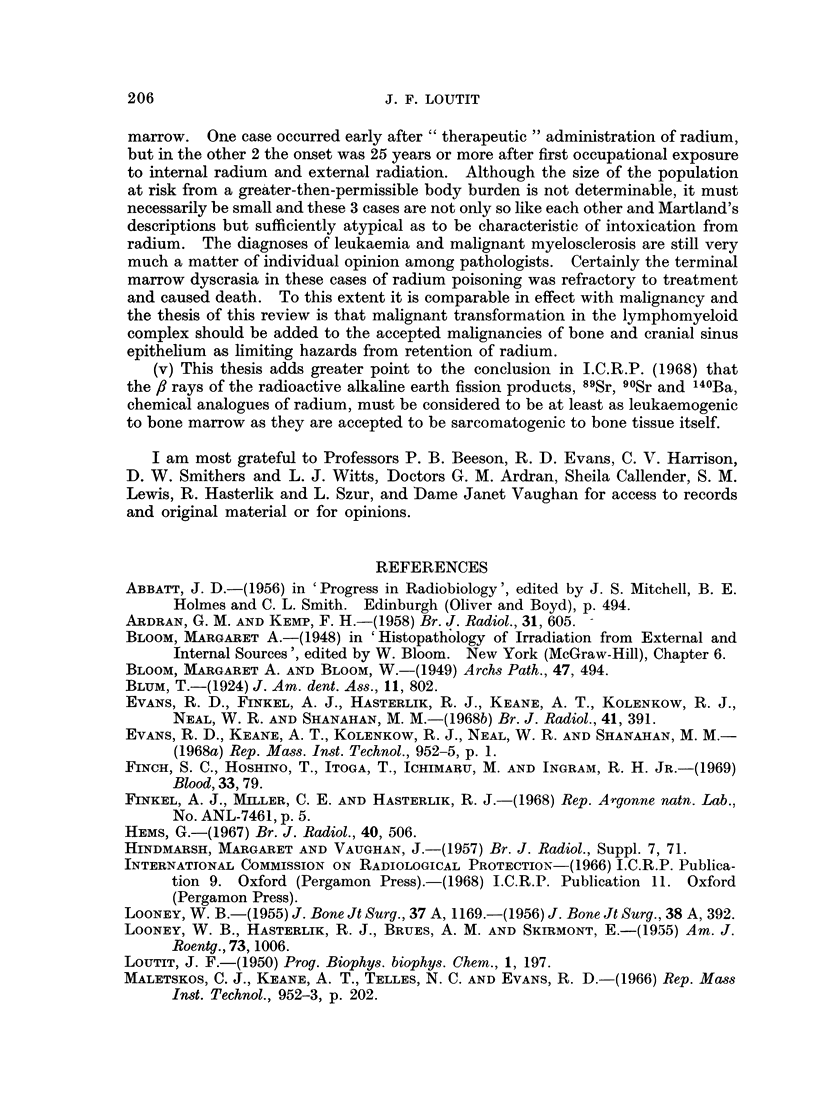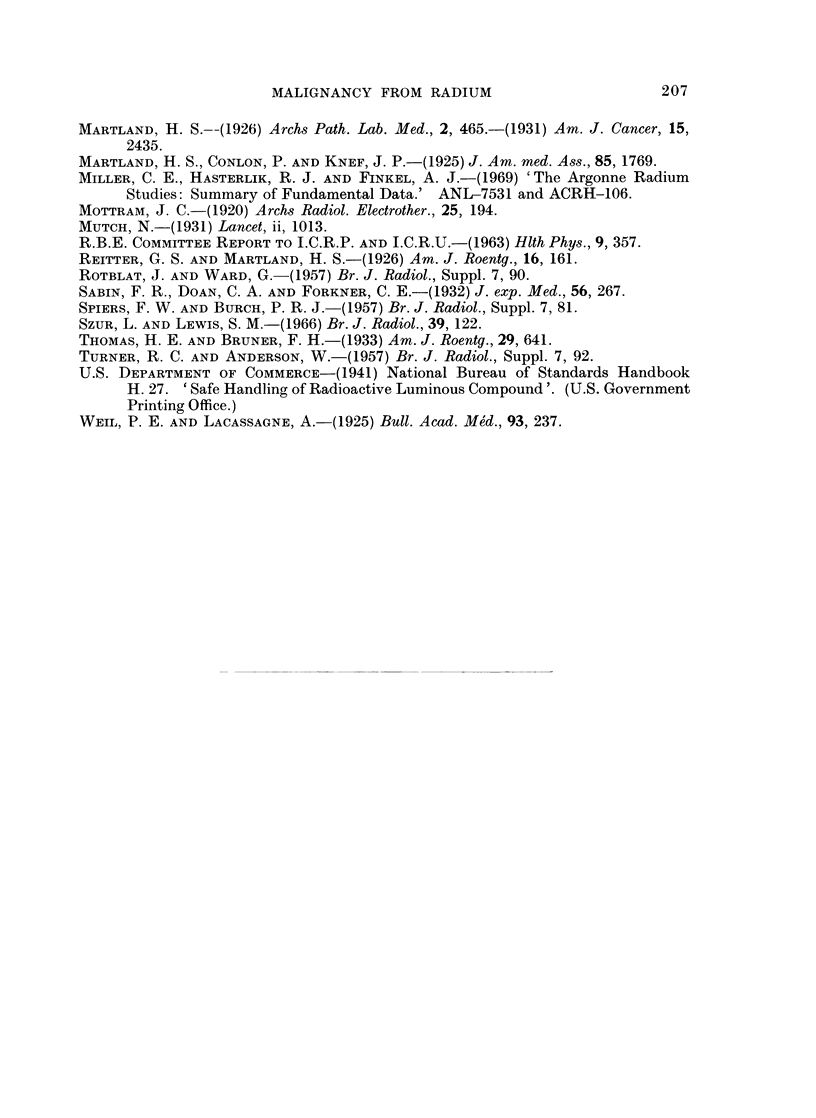# Malignancy from Radium

**DOI:** 10.1038/bjc.1970.23

**Published:** 1970-06

**Authors:** J. F. Loutit

## Abstract

**Images:**


### BRITISH JOURNAL OF CANCER

VOL. XXIV JUNE, 1970 NO. 2

### MALIGNANCY FROM RADIUM

J. F. LOUTIT

*External Staff of Medical Research Council*

*From the Medical Research Council Radiobiology Unit, Harwell, Didcot, Berks.*

Received for publication December 15, 1969

**SUMMARY.—Human experience of the toxicity of radium acts as a guide for the setting of occupationally permissible levels for radioactive nucleides, especially bone-seekers. Reviewing the published statements and photomicrographs in early reports especially those of Martland (1931) one can make a case that malignancy was induced in bone-marrow (leukaemia, malignant myelosclerosis) as well as in bone (osteosarcoma) by radium, especially with large doses. Three case reports of radium intoxication in Britons are noted as compatible with this suggestion, after revised interpretation in two of them.**

### RADIUM POISONING

WE still rely very heavily on rather inadequate human experience of the radiotoxicity of radium for regulating occupational exposure of man to internal contamination with radioactive substances.

The first report on radium poisoning is attributed to a dentist (Blum, 1924) who, in a discussion on osteomyelitis of the jaws, drew attention to a condition of " radium jaw " he had seen in luminizers, young women who painted dials with luminous paint. Luminizing had become a small industry during and after the First World War in U.S.A., Britain and continental Europe, but only from U.S.A. have substantial ill-effects in luminizers been recorded. This has been considered due to differences in practice. On the eastern side of the Atlantic application of luminous paint was by means of pens, in America till 1926 or thereabouts with brushes which the operators, mostly young women, " tipped " with their lips. This " tipping " thus led to ingestion of some paint, a mixture of scintillator, zinc sulphide, and radioactive salt, usually the sulphate of radium ($^{226}$Ra—T$\frac{1}{2}$ 1620y) or mesothorium I ($^{228}$Ra—T$\frac{1}{2}$ 5·7y) or a mixture thereof, with variable amounts of their radioactive decay products depending on the freshness of the isolation of the parent material. It may surprise the physician, accustomed to prescribe $BaSO_4$ in decagram amounts for barium meals, without fear of the highly chemically toxic barium ion being absorbed, that ingestion of milligram amounts of the vastly more insoluble ($\times$ 200) radium sulphate can lead to significant absorption. Experience shows, however, that retention of microgram amounts of radium in the body resulted even from quite short periods of this malpractice. Recently the surprisingly large figure of about 20% was obtained for absorption of radium ($^{224}$Ra—T$\frac{1}{2}$ 3·64d) sulphate as simulated luminous paint in normal, though elderly, human subjects (Maletskos, Keane, Telles and Evans, 1966): thus the appreciable retention of radium in dial painters is understandable, though the process of transport across the gut is still inexplicable. In the same experiments (Maletskos *et al.*) it was shown that thorium was not absorbed from the gut. Thus $^{228}$Th, the decay product of $^{228}$Ra in some luminous compounds, was an unlikely primary cause of intoxication.

The early cases of " radium jaw " were probably of secondary infection from the gums of jaw bones necrotic from the bombardment of osteocytes by ionizing particles from the radioactive elements in the bone mineral. Reviewing the reports of the late nineteen-twenties and early thirties, I wrote (Loutit, 1950), " The so-called osteitis or bone necrosis seems to occur in those more acute types of radium poisoning. Coincidentally or even in advance of the bone symptoms, there is an anaemia. Martland, Conlon and Knef (1925) noted it in luminizers and Reitter and Martland (1926) in a radio-chemist. While the peripheral blood suggested an aplastic anaemia such as had been observed by Mottram (1920) and Weil and Lacassagne (1925) the marrow was in fact hyperplastic. The photographs and photomicrographs of Martland (1931) are incontestable. In experimental animals both aplasia and hyperplasia of the marrow have been found, sometimes both together (Thomas and Bruner, 1933), aplasia in the ribs, vertebrae, etc., and hyperplasia in the shafts of the long bone."

These effects of radium in bone on the bone marrow are now largely ignored (*vide infra*).

### SARCOMA IN BONE

Martland (1931), who made many of the critical observations on the luminizers in New Jersey, was quick to observe that primary sarcoma of bone was a frequent consequence of incorporation of radium (and mesothorium) in bone and the phenomenon could be confirmed in experimental animals (Sabin, Doan and Forkner, 1932). These cancers appeared somewhat later than the bone necroses and anaemias: thus it soon became accepted that osteosarcoma was likely to be the limiting hazard. By 1941 the competent authorities, having evidence of death from malignant disease with terminal body burdens near to 1 $\mu$g., set permitted limits for the occupationally exposed as 0·1 $\mu$g. (U.S. Department of Commerce, 1941). It was this figure that was used as a yardstick for the calculations of comparable permissible body burdens of the newly derived radioactive bone-seeking isotopes produced in the then current Second World War programme in nuclear energy. This was a notable decision for 1941 and the figure is still acceptable in 1969.

The derivation of this permissible burden owed much to Professor Robley Evans of Massachusetts Institute of Technology, who, approaching the problem as a physicist, was concerned with the problems of physical dosimetry of these small quantities. He was later inspired to make the best possible ascertainment of the biological effects of intra-skeletal radioactivity by epidemiological survey of the population at risk. Determined efforts were made to trace all the ex-luminizers from the Eastern American States and in addition the net caught up many ex-radiochemists and ex-recipients of radium and thorium compounds, taken or injected under medical (*sic*) advice or sold as proprietary nostrums. A generation ago there had been a fashion to treat with radium many intractable diseases, *e.g.* syphilis, hypertension, gout, infectious polyarthritis, " muscular rheumatism ", leukaemia, pernicious and other anaemias, epilepsy and multiple sclerosis (Looney, Hasterlik, Brues and Skirmont, 1955; Mutch, 1931).

A similar survey has been under way in Chicago tapping Middle Western sources of ex-luminizers and patients (Hasterlik and his colleagues). These two major surveys are still far from complete but have yielded valuable information. The latest report from M.I.T. (Evans, Keane, Kolenkow, Neal and Shanahan, 1968*a*) relates to 496 cases studied either alive or *post mortem*. Two types of malignant tumour are specifically cited, the sarcoma arising in bone and carcinoma arising in epithelia closely adherent to bone, *e.g.* nasal sinuses, mastoid. These

FIG. 1.—Fig. 8 of Evans *et al.* (1968*a*) Burden time *v.* PRE, for dial painters and iatrogenic cases.

epithelia are within the limited range of the $\alpha$ particles of $^{226}Ra$ (40 $\mu$m) and decay products of $^{226}Ra$ and $^{228}Ra$ (up to 80 $\mu$m) from the subjacent mineral bone. In the M.I.T. series so far 37 tumours (28 sarcomas, 9 carcinomas) attributable to the contamination of bone have been observed. In Fig. 1 Evans and his colleagues plot survival since first exposure against latest body burden as pure radium equivalent (PRE): this measure is a means of handling cases of contamination with either $^{226}Ra$ alone or $^{226}Ra$ and $^{228}Ra$ together. Another parameter is the derived average dose in rads accumulated from the time of contamination till death or to the present time for those still living. Evans *et al.* take great pains to indicate that incidence of tumour is related to accumulated rads (CR) in a markedly non-linear way (Fig. 2), that is there is an effective threshold which justified the acceptance of a permissible body burden of 0·1 $\mu$g. $^{226}$Ra.

The Chicago group in their latest report (Finkel, Miller and Hasterlik, 1968) plot incidence of radium-induced malignancies (18 bone tumours and 12 carcinomas of skull and 3 cases of blood dyscrasia) versus current or preterminal radium burden (they were not bothered with $^{228}$Ra as a source) (Fig. 3). This graph gives an impression of linearity of dose-response. However, not only is the dose plotted logarithmically but the point at 0·17 $\mu$Ci is suspect. This maxillary

FIG. 2.—Fig. 17 of Evans *et al.* (1968*a*). The observed radiogenic tumour incidence, and some illustrative dose-response hypotheses. The solid circles are the observed values of tumour incidence, the lines the hypothetical-dose-responses, (2) being the threshold hypothesis which fits the data best.

" tumour " removed surgically from a patient who is still alive is regarded by some expert pathologists as not a carcinoma. If this one case is excluded the data are still reconcilable with the threshold hypothesis of Evans *et al.*, the threshold being about 0·5 $\mu$g. PRE.

Another feature of the report of Finkel *et al.* (1968) is the observation of a greater than expected mortality in luminizers from tumours of the central nervous system (× 20) and lungs (× 5). Though these figures require affirmation, this indicates a need for not analysing solely for specific tumours such as osteosarcoma and carcinoma of nasal sinuses.

Reviewing earlier progress reports Hems (1967) in this country has made a case for a linear relation between incidence of tumour and initial radium content. In principle a linear dose-response is accepted as a working rule in radiation-protection (International Commission on Radiological Protection, 1966), since at low doses and low dose rates current radiobiological theory would indicate its probability (R.B.E. Committee, 1963). In fact Hems made some errors of expression, of fact and of interpretation of the American data which elicited a strongly worded rebuttal of the errors (Evans, Finkel, Hasterlik, Keane, Kolenkow, Neal and Shanahan, 1968*b*). I shall not fall into the same trap now of trying to extract information from the already compressed reports of others. While accepting for practical purposes the thesis of an effective threshold of body burden, like Hems and others I feel intuitively that a linear relationship has not yet been excluded in spite of persuasive statistical argument by Evans *et al.* (1968*a*). Examination of Fig. 1 (and Fig. 4 of Evans *et al.*, 1968*a*) shows that the 24 cases in the 10–100 μCi band are a special group with a much shortened expectation of life: whether they died of malignant disease or not, it is near-certain

FIG. 3.—Fig. 3 of Finkel *et al.* (1968). Incidence of radium-induced malignant tumours and blood dyscrasias in the ANL–ACRH series plotted against current or preterminal radium burdens in μCi, September 1967.

that radiation was the fundamental cause. Furthermore, the cases within the 1–10 μCi band are differently distributed between live and dead from those in the 0·1–1 μCi band: additionally at the upper levels within this group, as with the cases containing 10–100 μCi, there is life-shortening. It seems to the simple minded like me that these two groups cannot be validly compared with the others less contaminated in which, be it noted, no individual data are available. I conclude the material is not analysable without access to the detailed records and probably not till the whole population is dead. Even the whole population so far surveyed by M.I.T. and Chicago together (~ 700 people) is a small one by epidemiological standards and contains some bias of which the surveyors but not third parties are aware. A British population of ex-luminizers with smaller radium burdens is under surveillance by Boyd and Vennart according to Hems (1967).

DYSCRASIA OF BONE MARROW

The report from Chicago (Finkel *et al.*, 1968) confirms the earlier observations of Martland (1926) that in their series some of the early deaths were attributed to " blood dyscrasias ". Leaving aside one reported case of chronic lymphatic leukaemia, which arose in their series of " cases studied ", because this type of leukaemia is not generally accepted as radiation-induced (Finch, Hoshino, Itoga, Ichimaru and Ingram, 1969), one notes one case of splenic leukaemia (allegedly synonymous with chronic granulocytic leukaemia) in the " cases studied ", another certified as such and 2 cases of " aplastic anaemia " certified among the " not studied " luminizers and another " aplastic anaemia " and a " panmyelosis " in medical cases treated with radium. In the last 2 cases and the one accredited case of splenic leukaemia measurements indicated a chronically retained radium burden of 10 $\mu$Ci or more. There is a strong indication, therefore, that at least with substantial contamination the bone marrow is affected.

In recent years any effect on the bone marrow has been discounted because of the limited range of $\alpha$ particles of Ra (40 $\mu$m) in the marrow cavities of trabecular bone 500–1000 $\mu$m across (I.C.R.P., 1968). Instead attention has been concentrated on endosteal progenitive cells. Both the clinical and experimentally produced tumours from $^{226}$Ra appear to be endosteal in origin (I.C.R.P., 1968). The clinical factors in the report of Finkel *et al.* (1968) suggest, however, that many of these sarcomas are atypical often being recorded as fibrosarcomata and frequently in unusual sites. In view of the newer data it is proper to keep an open mind concerning radiosensitive tissues in bone. We must not neglect marrow and we must remember that it has been assumed so far by most of us that haemopoietic stem cells are randomly distributed in marrow: if in fact the distribution is not random and there is a concentration at the periphery of the trabecular net, the marrow is at greater risk than hitherto calculated.

With this in mind I have studied again after a lapse of 20 years Martland's original descriptions of his early material and the following are quotations from Martland (1931) except for my italicized parentheses.

(1) " From 1922 to 1928, 13 deaths occurred which I have designated as early cases. The cases showed during life a clinical picture quite different from that in cases which developed later " (*but see* (4)). " They were characterized by the presence of jaw necrosis and the development of anemias. Most of these cases occurred 4 and 6 years after the girls left their employment as dial painters." (*N.B.* 4 *were chemists!*)

(2) " A leukopenic anemia of the regenerative type (red marrow) developed. This anemia when once established resisted all modern forms of treatment and usually proved rapidly fatal."

(3) " In the early cases, mesothorium, which is chemophysically and physiologically more active than radium, predominates."

(4) (*Then in describing* 5 *fatal later cases of osteosarcoma* 1924–1931.)

" Case 2. Necropsy—a profound anemia was present. The yellow marrow of the femurs was replaced by a dark red, apparently regenerating marrow. . . . The sections showed a regenerative, hyperplastic marrow similar to that described in other cases of radium poisoning."

" Case 3. Necropsy—the marrow of the right femur was dark mottled red color and showed many greyish white areas of radiation osteitis (*? in cancellous*

*parts*) measuring 1 to 2 cm. . . . (Fig. 4). Sections made from the femur and vertebra showed a regenerating marrow of the megaloblastic type with many primitive cells. Mature cells of the granulocyte series were scant, except for the presence of innumerable eosinophil myelocytes. Megakaryocytes appeared to be abundant. In many of the marrow cavities this hyperplastic marrow was beginning to be replaced by a very cellular fibroblastic growth containing many eosinophil myelocytes, plasma cells and lymphocytes (Fig. 5). Mitotic figures in these areas were common and such areas were distinguished only with great difficulty from sarcoma. In other areas, especially those which grossly appeared as hard, greyish areas of radiation osteitis, this fibroblastic replacement of the original hyperplastic marrow was distinctly acellular. Here the marrow had been replaced by more or less dense fibroblastic acellular tissue."

"Case 4. Necropsy—the calvarium was hard and 'ivory-like' and showed hardly any cancellous bone. An occasional oval, expansive area in the inner table of the skull in the parietal region was seen. On section a small amount of red cancellous bone was observed in these areas with thinning and erosion of the inner table (radiation osteitis) (Fig. 6). The bodies of the vertebrae showed occasional greyish white areas of radiation osteitis in the cancellous bone. The marrow in the middle of the femur was red and hyperplastic. Sections from the femur and vertebra showed a regenerating marrow of the megaloblastic type with many primitive cells resembling hemocytoblasts. In many of the marrow cavities this hyperplastic marrow was beginning to be replaced by a very cellular fibroblastic growth. . . . So cellular were some of these areas that they could not be distinguished from sarcoma. . . . Sections . . . of the skull showed that the marrow spaces in the inner half of the calvarium were filled with the same hyperplastic marrow containing many eosinophil myelocytes with large numbers of osteoclasts causing erosion of bone."

"Case 5. Necropsy—the bone marrow in the middle of the femurs was a deep, intense red color. The marrow in the vertebrae was deep red in color and showed many lighter areas of radiation osteitis. Sections from the bone marrow showed the typical hyperplastic marrow seen in the other cases, with many areas of radiation osteitis scattered throughout the marrow."

(5) (*Later in a general discussion of radiation osteitis.*)

(*a*) " In every one of the radioactive dial painters whom I have autopsied . . . the marrow of the femurs was dark red throughout, and the lesion more pronounced than that seen in the most characteristic case of pernicious anemia. Histologically the marrow showed an astonishing picture, quite unlike that seen in any other disease. The general architecture, structure and landmarks were entirely obscured by the extreme hyperplasia, with a packing of immature and primitive cells. The marrow spaces were so filled that in the smaller a distinct widening and increase took place. In the cancellous portions of the calvarium this sometimes was so marked as to produce localized areas of apparent rarefaction in the roentgenograms, causing the so-called skull lesions in some cases. Some 60% of the cells in well packed areas were very large, 12 to 15 to 20 microns in diameter, with large vesicular nuclei containing one to three or more nucleoli. There was a distinct nuclear limiting membrane with condensation of nuclear chromatin at its edges. The cytoplasm was dull, bluish grey (hematoxylin–eosin), smooth, and glassy, and contained no granules. The cells contained no hemoglobin (Fig. 7). . . . Mixed with these predominating primitive cells were many megaloblasts, . . . many in mitosis. Normoblasts of all varieties were present. . . . The only cells of the granulocytic series were innumerable eosinophil myelocytes. . . . Lymphocytes were not present, nor were other cells of the lymphoblastic series. Megakaryocytes were usually abundant."

(*b*) " After this hyperplastic irritative marrow has developed over various parts of the skeleton, the lesion starts to subside or heal in patchy areas. This is essentially a replacement fibrosis. . . . In the beginning the reaction is a very cellular one. Many of these can be differentiated from sarcoma only with great difficulty. . . . It is important to note that all stages of this radiation osteitis may be seen in a single bone " (Fig. 8 and 9).

This very clear description combined with the illustrations makes it clear that this is not aplastic anaemia in the strict sense of the term. It is as Martland emphasized a hyperplastic state of the marrow. He rules out haemolysis as a cause since haemosiderin was not in excess. He ruled out true Addisonian pernicious anaemia, though obviously he is not clear on the more recently accepted differentiation of megaloblastic and normoblastic hyperplasia. Although there is no mention of polycthaemia, the descriptions of the marrow are very reminiscent of those of a late stage of polycythaemia rubra vera with supervening myelosclerosis (Szur and Lewis, 1966). This myelosclerosis may be simple or malignant in histological appearance and leukaemic transformation is another well-recognized consequence of the polycythaemic lesion. Martland's description is to me that of malignant myelosclerosis, perhaps even in some cases of erythraemic myelosis (*i.e.* leukaemia).

Since Martland's description and interpretation of the gross and microscopic pathology of the marrow, little mention has been made of the bone marrow in subsequent reports. The interest has been concentrated on measurements of radium, roentgenographic appearances (Looney *et al.*, 1955), development of osteosarcomas and cranial carcinomas and the interrelation between dose and effect. Even massive compilations of data, (Miller, Hasterlik and Finkel, 1969) give little or no information about the pathology of bone marrow except at the locus of interest, the tumour.

It may be that Martland's vivid description applies only to cases exposed predominantly to mesothroium—$^{228}$Ra—but a total of 7 " leukemias and other blood dyscrasias " are listed in the most recent summary of cases from Chicago (Finkel *et al.*, 1968) where mesothorium was not involved in significant amount. Looney (1956), it is true, does mention two cases (R–24 and R–43) where the description does not tally with Martland's. Earlier, he (1955) shows illustrations which seem to indicate profound but simple myelosclerosis.

---

### EXPLANATION OF PLATES

FIG. 4.—Fig. 10 of Martland (1931) with caption.
FIG. 5.—Fig. 30 of Martland (1931) with caption.
FIG. 6.—Fig. 44 of Martland (1931) with caption.
FIG. 7.—Fig. 35 of Martland (1931) with caption.
FIG. 8.—Fig. 39 and 40 of Martland (1931) with caption.
FIG. 9.—Fig. 41 of Martland (1931) with caption.
FIG. 10 and FIG. 11.—Fig. 3 from Abbatt (1956).
FIG. 12.—Bone marrow from Abbatt's case. H and E. × 1470.
FIG. 13.—Bone marrow from Abbatt's case, reticulin stain × 200.
FIG. 14.—Radiogram of skull—Case 2 of Ardran and Kemp (1958).

Fig. 10. Case 3. Portions of the Femur, Humerus, and Spine Showing Multiple Scattered Areas of Radiation Osteitis.

These might be mistaken for metastases. However, in primary bone sarcoma metastases to other bones are rare. Histologically they showed the typical areas of osteitis described in this paper.

4

Fig. 30. Case 3. Radiation Osteitis. Microscopic Appearance of Gross, Lesions Shown in Fig. 29.

All three successive stages of radiation osteitis occurring in the dial painters may be noted in the same section. The first stage of hyperplastic, irritative marrow (A); the second stage of cellular fibroblastic replacement in which areas the sarcomas are likely to develop (B); and the final or healing stage of acellular fibrosis with decalcification (C). × 42.

In experimental animals given very substantial doses of $^{226}Ra$ Bloom (1948) reported gelatinous fibrous marrow in trabecular bone but " with lower doses the marrow of the shaft was hyperplastic rather than depleted ". At still lower doses Bloom and Bloom (1949) record overgrowth of trabecular bone with " devitalization " merging with dense gelatinous marrow but " on the whole it is hemopoietic ".

### CASE REPORTS IN BRITAIN

The one case of radium-poisoning with which I have had some personal contact was briefly reported for the haematological features by Abbatt (1956) and for radiation dosimetric features by Hindmarsh and Vaughan (1957), Rotblat and Ward (1957), Turner and Anderson (1957) and Spiers and Burch (1957). The patient was a radium chemist aged 74 who died in 1954 from " acute myeloid leukaemia " (erythromyelosis), 15 years after retirement from an occupation which must have resulted in marked exposure to external $\gamma$-irradiation as well as leading to a terminal body burden of radium estimated as 0·3 to 0·5 $\mu$Ci.

Abbatt (1956) records the detail of routine blood counts over the last 20 years of his occupational exposure. No polycythaemia was recorded and " apart from a marked leucopenia which was present in 1919 and a mild degree of anaemia in 1936 there were few marked fluctuations ". In the rapid terminal illness of only a few weeks there was anaemia and leucopenia with blast cells and erythrocyte precursors in the peripheral blood. A marrow biopsy yielded cellular hyperplastic marrow which in stained films indicated predominance of erythropoiesis " many abnormal megaloid erythroblasts being present. Leucopoiesis was defective with an excess of myeloblasts " (Professor J. V. Dacie). Abbattt illustrates the areas of osteolysis in radiograms of skull and humerus characteristic of radium retention (Fig. 10 and 11).

The post-mortem report by Professor C. V. Harrison includes the record of hyperplastic red marrow in sternum, pelvis, ribs and spine, upper halves of humerus and femur with speckled red and yellow marrow in the lower half of femur, yellow marrow in tibia and metacarpals and a remarkable appearance in the calvarium with innumerable small, red marrow filled cavities of osteoporosis, mostly under 5 mm. diameter and more easily visible from the inner surface. The histological report describes hyperplastic compact cellular marrow in sternum, iliac crest, 5th lumbar vertebral body and femoral shaft " showing myelopoiesis and erythropoiesis, the former predominating. Primitive cells are unduly numerous. Plasma cells are also relatively frequent." My personal view was to be impressed more with the density and range of erythroid cells and an apparent lack of late forms in the neutrophil granulocyte series plus the abundance of eosinophil myelocytes (Fig. 12). Concerning the skull the report states: " Focus of osteoporosis. The outer table is normal: the inner table is replaced by cancellous bone enlarging the marrow cavity. At this point the marrow is entirely cellular. Away from this focus the marrow is largely fatty. There appears to be association between the osteoporosis and the localized marrow hyperplasia." Notably there is no record of fibrosis in marrow and I could find only very occasional narrow belts in apposition to a few trabeculae of the L.5 vertebral body. In sections of marrow " the amount of reticulin is greater than normal but not enormously so " (C. V. Harrison—Fig. 13). Notably also there was little extramedullary haematopoiesis, perhaps a little in a 263 g. siderotic spleen. (N.B. Recent transfusions of packed red cells from stored blood.)

Another similar case has been recorded in a British journal (case 2 of Ardran and Kemp, 1958). A man of 53 employed for about 25 years as a radium technician was investigated in Hove General Hospital in 1947, and found to emit $\gamma$-rays corresponding to about 0·3 $\mu$g. of radium decay products (*i.e.* perhaps a body content of 0·5 $\mu$g. Ra). In the Radcliffe Infirmary, Oxford, a 2-year history of tiredness, followed later by pain in the back with increasing deformity, was obtained. He manifested marked kyphosis plus anaemia. A radiogram of the spine showed profound decalcification and collapse of vertebral bodies. Radiograms of the pelvis and skull were interpreted at the time as myelomatosis. Indeed, the radiogram of skull is typical of radium osteolysis (Fig. 14) and the report on the marrow by Professor L. J. Witts (quoted by Ardran and Kemp, 1958) notes the absence of characteristic myeloma cells. The precision of the diagnosis of myeloma is thus dubious. Unquestionably a myelosis was present, so that as Ardran and Kemp state " the resulting radiographic picture may simulate myelomatosis or osteoporosis ".

Reviewing this case in 1969 I incline to the myelosis being leukaemic: (*a*) primitive cells, " blasts " (4%) and " reticulum " (not rectum, as printed) cells (9%) were listed among the leucocytes (6500 mm.$^{-3}$) of peripheral blood. My own interpretation of the existing film is—Very unequal spreading: cells mostly in " tail ". In the body of the films granulocytes (24%) appear normal; lymphocytes (76%) are mostly large and about 10% are definitely primitive with grey-blue opaque cytoplasm and nuclei with indistinct chromatin pattern sometimes containing a few small nucleoli. A moderate number of smear cells present. The marrow smear though acellular was recorded as markedly abnormal with 55% lymphocytes and 19% lymphoblasts: this film also is still existent and according to my interpretation probably represents a tap of peripheral blood only. There is no further evidence from examination post-mortem.

Case 1 of Ardran and Kemp (1958) was a man in chronic ill health treated with " German Radium Salt " injections about 2 years before his death from supposed " aplastic anaemia ". His terminal symptoms of anaemia started several months only before his death. An unconfirmed estimate of his radium burden derived from one of many sources of information and quoted as from the Clarendon Laboratory, Oxford, was 4–5 $\mu$g. Re-examination of the records identifies another unfortunate misprint in Ardran and Kemp's publication. Professor W. G. Barnard reporting on the autopsy said: " The marrow of the right femur was examined. The marrow throughout the length of the shaft was hyperplastic but there was no hyperplasia of the ends of the bone "—not that the femoral marrow was hypoplastic. The Registrar in another place says, " The long bones were full of red marrow ". A report on the sections (which are no longer available) says curtly—marrow, normoblastic hyperplasia. Indeed this alters the diagnosis of aplastic anaemia and makes the case compatible with Martland's regenerative leucopenic anaemia. Numerous reports on the peripheral blood count support such a diagnosis, perhaps aleukaemic leukaemia with leucoerythroblastosis. Within the terminal few months 3 hospitals recorded anisocytosis and poikilocytosis of red cells, often marked. Reticulocytes varied from 0·4 to 2·2%. Normoblasts were several times reported in the peripheral blood, *e.g.* 756 mm.$^{-3}$ 2 months before death (with 900 mm.$^{-3}$ primitive granular cells) and 43 per

100 leucocytes a month later. The red cell volume was up to 130 $\mu m^3$. The one sternal puncture probably also tapped peripheral blood for the 2 counts are not significantly different.

| | Blood | Sternum |
|---|---|---|
| Myeloblast | 2 | — |
| Neutrophil myelocyte | 1 | 4 |
| Neutrophil young form | — | 2 |
| Neutrophil band form | 13 | 18 |
| Neutrophil segmented | 25 | 12 |
| Lymphocyte | 24 | 22 |
| Monocyte | 17 | 25 |
| Normoblast orthochromatic | 18 | 17 |

These films still exist and on examination are much as reported. The monocytes appear to be rather juvenile with gradations to rare (2%) blast cells. During the terminal illness radiograms of tibia and fibula, radius and ulna, were reported as not abnormal and indeed even with hindsight any characteristic lesions of radium retention are most dubious. From the evidence post-mortem one must conclude that the marrow of this man was not aplastic but frustrated.

To recapitulate—these 3 British cases are all instances of a bone marrow dyscrasia and all may well be classed as aleukaemic leukaemia. Case 1 of Ardran and Kemp (1958) is one of sub-acute intoxication following retention of radium for a few years only. Regenerative leukopenic anaemia (Martland, 1931), ? aleukaemic leukaemia, was the sequel. The other 2 cases are long delayed cases of occupational disease in which external irradiation and chronic internal retention of radium were both involved. For one of these 2 cases an acceptable diagnosis is erythroleukaemia, for the other without data post-mortem no specific title can be given.

### CONCLUSIONS

The limiting hazard from internally retained $^{226}Ra$ (and $^{228}Ra$ and $^{224}Ra$) acquired occupationally or aforetimes as therapy has been accepted for a generation as osteosarcoma.

In the U.S.A. surveys have demonstrated additionally in the last decade that carcinoma of epithelia closely applied to bone in the cranial air passages is another common terminal event: there is a suggestion that other neoplastic conditions, *e.g.* in the C.N.S., may be increased.

In the present review the following points are made:

(i) The M.I.T. survey in the eastern States indicates that subjects with substantial body burdens, *e.g.* 5–100 $\mu$Ci, have considerable shortening of life-span but the associated pathology has not been clarified.

(ii) In the Chicago survey some 7 cases of bone marrow dyscrasia have been reported amongst subjects in this class.

(iii) The classical reports of Martland *et al.* (1925) and Martland (1926, 1931), when the toxic nature of retained radium was first uncovered, describe very clearly a "regenerative leucopenic anemia" in such cases: this syndrome has features of atypical (aleukaemic) leukaemia or myelosclerosis or both.

(iv) Three cases of radium poisoning reported from Britain all appear to be compatible with a diagnosis of acute aleukaemic leukaemia, a short terminal illness with refractory anaemia, leucopenia associated with primitive nucleated cells in the peripheral blood and at autopsy on 2 of them, hyperplastic red bone marrow. One case occurred early after " therapeutic " administration of radium, but in the other 2 the onset was 25 years or more after first occupational exposure to internal radium and external radiation. Although the size of the population at risk from a greater-then-permissible body burden is not determinable, it must necessarily be small and these 3 cases are not only so like each other and Martland's descriptions but sufficiently atypical as to be characteristic of intoxication from radium. The diagnoses of leukaemia and malignant myelosclerosis are still very much a matter of individual opinion among pathologists. Certainly the terminal marrow dyscrasia in these cases of radium poisoning was refractory to treatment and caused death. To this extent it is comparable in effect with malignancy and the thesis of this review is that malignant transformation in the lymphomyeloid complex should be added to the accepted malignancies of bone and cranial sinus epithelium as limiting hazards from retention of radium.

(v) This thesis adds greater point to the conclusion in I.C.R.P. (1968) that the $\beta$ rays of the radioactive alkaline earth fission products, $^{89}$Sr, $^{90}$Sr and $^{140}$Ba, chemical analogues of radium, must be considered to be at least as leukaemogenic to bone marrow as they are accepted to be sarcomatogenic to bone tissue itself.

I am most grateful to Professors P. B. Beeson, R. D. Evans, C. V. Harrison, D. W. Smithers and L. J. Witts, Doctors G. M. Ardran, Sheila Callender, S. M. Lewis, R. Hasterlik and L. Szur, and Dame Janet Vaughan for access to records and original material or for opinions.

### REFERENCES

ABBATT, J. D.—(1956) in 'Progress in Radiobiology', edited by J. S. Mitchell, B. E. Holmes and C. L. Smith. Edinburgh (Oliver and Boyd), p. 494.

ARDRAN, G. M. AND KEMP, F. H.—(1958) *Br. J. Radiol.*, **31**, 605.

BLOOM, MARGARET A.—(1948) in 'Histopathology of Irradiation from External and Internal Sources', edited by W. Bloom. New York (McGraw-Hill), Chapter 6.

BLOOM, MARGARET A. AND BLOOM, W.—(1949) *Archs Path.*, **47**, 494.

BLUM, T.—(1924) *J. Am. dent. Ass.*, **11**, 802.

EVANS, R. D., FINKEL, A. J., HASTERLIK, R. J., KEANE, A. T., KOLENKOW, R. J., NEAL, W. R. AND SHANAHAN, M. M.—(1968*b*) *Br. J. Radiol.*, **41**, 391.

EVANS, R. D., KEANE, A. T., KOLENKOW, R. J., NEAL, W. R. AND SHANAHAN, M. M.—(1968*a*) *Rep. Mass. Inst. Technol.*, 952–5, p. 1.

FINCH, S. C., HOSHINO, T., ITOGA, T., ICHIMARU, M. AND INGRAM, R. H. JR.—(1969) *Blood*, **33**, 79.

FINKEL, A. J., MILLER, C. E. AND HASTERLIK, R. J.—(1968) *Rep. Argonne natn. Lab.*, No. ANL-7461, p. 5.

HEMS, G.—(1967) *Br. J. Radiol.*, **40**, 506.

HINDMARSH, MARGARET AND VAUGHAN, J.—(1957) *Br. J. Radiol.*, Suppl. 7, 71.

INTERNATIONAL COMMISSION ON RADIOLOGICAL PROTECTION—(1966) I.C.R.P. Publication 9. Oxford (Pergamon Press).—(1968) I.C.R.P. Publication 11. Oxford (Pergamon Press).

LOONEY, W. B.—(1955) *J. Bone Jt Surg.*, **37** A, 1169.—(1956) *J. Bone Jt Surg.*, **38** A, 392.

LOONEY, W. B., HASTERLIK, R. J., BRUES, A. M. AND SKIRMONT, E.—(1955) *Am. J. Roentg.*, **73**, 1006.

LOUTIT, J. F.—(1950) *Prog. Biophys. biophys. Chem.*, **1**, 197.

MALETSKOS, C. J., KEANE, A. T., TELLES, N. C. AND EVANS, R. D.—(1966) *Rep. Mass Inst. Technol.*, 952–3, p. 202.

Martland, H. S.--(1926) *Archs Path. Lab. Med.*, **2**, 465.—(1931) *Am. J. Cancer*, **15**, 2435.
Martland, H. S., Conlon, P. and Knef, J. P.—(1925) *J. Am. med. Ass.*, **85**, 1769.
Miller, C. E., Hasterlik, R. J. and Finkel, A. J.—(1969) 'The Argonne Radium Studies: Summary of Fundamental Data.' ANL–7531 and ACRH–106.
Mottram, J. C.—(1920) *Archs Radiol. Electrother.*, **25**, 194.
Mutch, N.—(1931) *Lancet*, ii, 1013.
R.B.E. Committee Report to I.C.R.P. and I.C.R.U.—(1963) *Hlth Phys.*, **9**, 357.
Reitter, G. S. and Martland, H. S.—(1926) *Am. J. Roentg.*, **16**, 161.
Rotblat, J. and Ward, G.—(1957) *Br. J. Radiol.*, Suppl. 7, 90.
Sabin, F. R., Doan, C. A. and Forkner, C. E.—(1932) *J. exp. Med.*, **56**, 267.
Spiers, F. W. and Burch, P. R. J.—(1957) *Br. J. Radiol.*, Suppl. 7, 81.
Szur, L. and Lewis, S. M.—(1966) *Br. J. Radiol.*, **39**, 122.
Thomas, H. E. and Bruner, F. H.—(1933) *Am. J. Roentg.*, **29**, 641.
Turner, R. C. and Anderson, W.—(1957) *Br. J. Radiol.*, Suppl. 7, 92.
U.S. Department of Commerce—(1941) National Bureau of Standards Handbook H. 27. 'Safe Handling of Radioactive Luminous Compound'. (U.S. Government Printing Office.)
Weil, P. E. and Lacassagne, A.—(1925) *Bull. Acad. Méd.*, **93**, 237.